# Supplementary material for: Optimized Magnetization Distribution in Body-Centered Cubic Lattice-Structured Magnetoelastomer for High-Performance 3D Force–Tactile Sensors
Source: Sensors (Basel). 2025 Apr 5;25(7):2312. doi: 10.3390/s25072312 (PMC11990970; doi:10.3390/s25072312)
Supplement: Supplementary file 1 [file sensors-25-02312-s001.zip › sensors-3533730-supplementary.pdf]

Supporting Information

Optimized Magnetization Distribution in Body-Centered Cubic Lattice-Structured Magnetoelastomer for High-Performance 3D Force–Tactile Sensors

Hongfei Hou [1,2,3], Ziyin Xiang [2,3], Chaonan Zhi [2,3], Haodong Hu [2,3], Xingyu Zhu [1,2,3], Baoru Bian [2,3], Yuanzhao Wu [2,3], Yiwei Liu [2,3], Xiaohui Yi [2,3,*], Jie Shang [2,3,*] and Run-Wei Li [2,3,4,*]

1 School of Materials Science and Chemical Engineering, Ningbo University, Ningbo 315211, China; houhongfei@nimte.ac.cn (H.H.); zhuxingyu@nimte.ac.cn (X.Z.)
2 CAS Key Laboratory of Magnetic Materials and Devices, Ningbo Institute of Materials Technology and Engineering, Chinese Academy of Sciences, Ningbo 315201, China; xiangziyin@nimte.ac.cn (Z.X.); zhichaonan@nimte.ac.cn (C.Z.); huhaodong@nimte.ac.cn (H.H.); bianbr@nimte.ac.cn (B.B.); wuyz@nimte.ac.cn (Y.W.); liuyw@nimte.ac.cn (Y.L.)
3 Zhejiang Province Key Laboratory of Magnetic Materials and Application Technology, Ningbo Institute of Materials Technology and Engineering, Chinese Academy of Sciences, Ningbo 315201, China
4 College of Materials Science and Opto-Electronic Technology, University of Chinese Academy of Sciences, Beijing 100049, China
* Correspondence: yixiaohui@nimte.ac.cn (X.Y.); shangjie@nimte.ac.cn (J.S.); runweili@nimte.ac.cn (R.-W.L.)

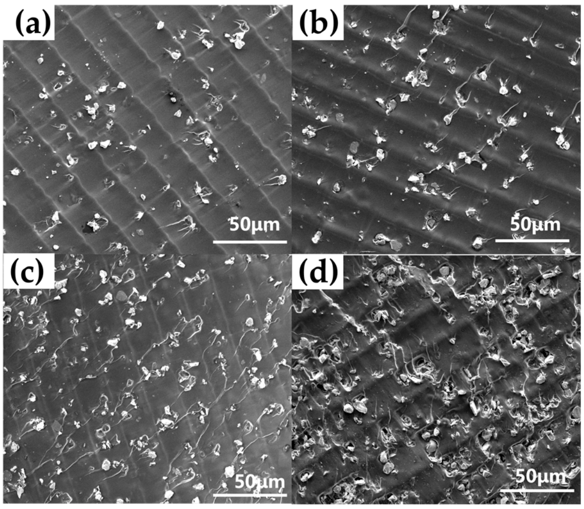

Figure S1. Scanning electron microscope (SEM)images of cross sections of samples with different magnetic particle contents. (a)10wt% (b)20wt% (c)20wt% (d)40wt%

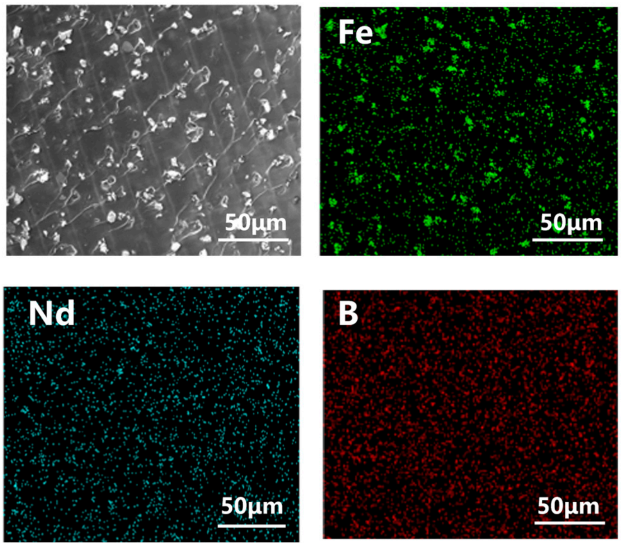

Figure S2. Scanning electron microscope (SEM)images and the distribution maps of Fe, Nd, and B elements for the sample containing 30wt % magnetic particles.

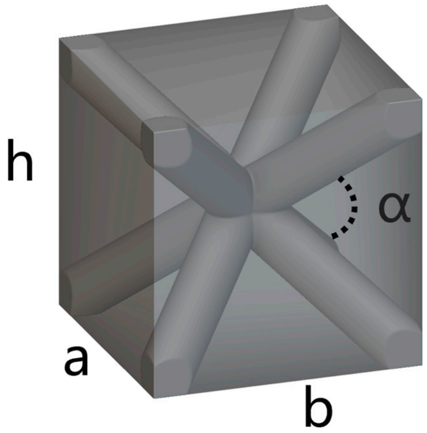

Figure S3. BLSM unit parameters

Table S1. BLSM structure angle and parameters (mm).

Structural angle （α）	60°	70.6°	90°
lattice parameters （a b h）	（3.1 3.1 2.5）	（2.5 2.5 2.5）	（1.77 1.77 2.5）
$\alpha = 2\, arctan\left(\frac{h}{\sqrt{a^2+b^2}}\right)$			

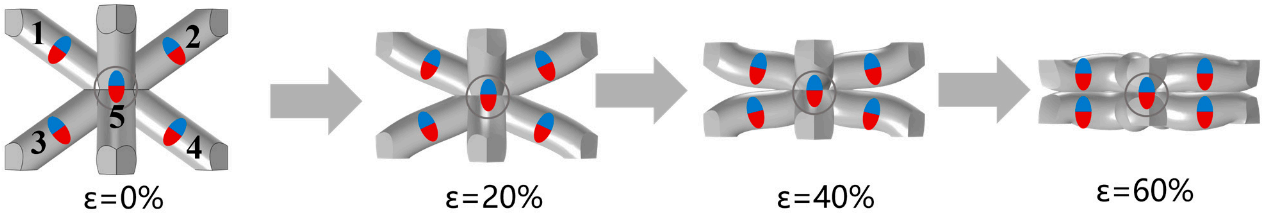

Figure S4. The magnetization direction of each part of the BLSM unit in different compression states.

Table S2. The vector of the magnetization direction in each part under different compression states.

<table>
<tr><th>Compression</th><th>magnetization direction （1, 4）</th><th>magnetization direction （2, 3）</th><th>magnetization direction （5）</th></tr>
<tr><td>0%</td><td>(-1, 0， $-\sqrt{2}$)</td><td>(1, 0, $-\sqrt{2}$)</td><td rowspan="4">（0, 0, -1）</td></tr>
<tr><td>20%</td><td>(-2, 0, $-3\sqrt{2}$)</td><td>(2, 0, $-3\sqrt{2}$)</td></tr>
<tr><td>40%</td><td>(-1, 0, $-3\sqrt{2}$)</td><td>(1, 0, $-3\sqrt{2}$)</td></tr>
<tr><td>60%</td><td>(0, 0， -1)</td><td>(0, 0, -1)</td></tr>
</table>

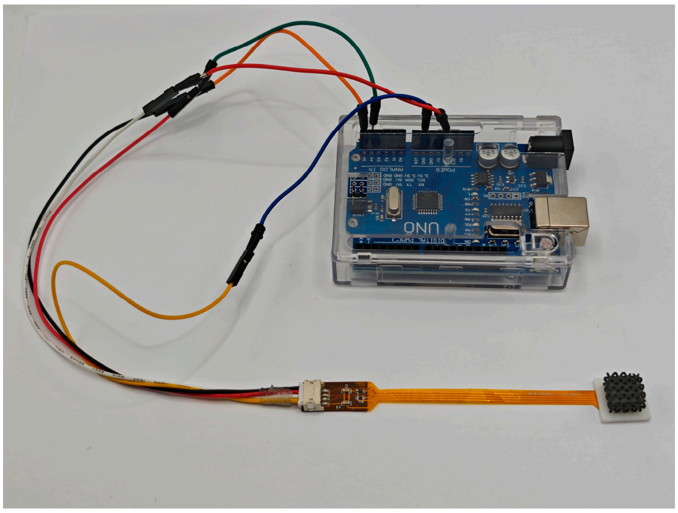

Figure S5. Flexible magnetic tactile sensor.

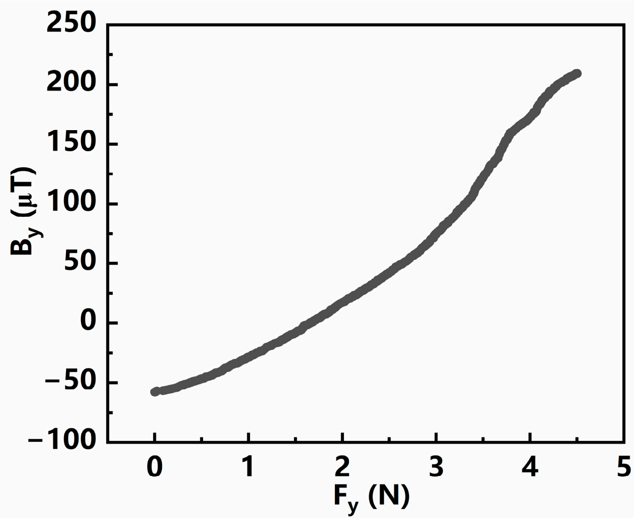

Figure S6. The magnetic response of the tangential force parallel to the Y direction.
